# *N*-[2-Chloro-6-(4-chloro-6-methoxy­pyrimidin-2-ylsulfan­yl)benz­yl]-3,4-dimethyl­aniline

**DOI:** 10.1107/S1600536809041026

**Published:** 2009-10-17

**Authors:** Weijun Fu, Mei Zhu, Dongfeng Hong

**Affiliations:** a College of Chemistry and Chemical Engineering, Luoyang Normal University, Luoyang 471022, People’s Republic of China

## Abstract

In the title mol­ecule, C_20_H_19_Cl_2_N_3_OS, the dihedral angle between the two benzene rings is 79.3 (7)°. The 4-chloro-6-methoxy­pyrimidine group is rotationally disordered over two sites by approximately 180°, the ratio of the refined occupancies being 0.6772 (15):0.3228 (15). Both disorder components of disorder are involved in intra­molecular N—H⋯N hydrogen bonds.

## Related literature

For the biological functions of pyrimidine derivatives, see: Joffe *et al.* (1989 ▶); Petersen & Schmidt (2003 ▶); Blum (2001 ▶); Gompper *et al.* (2004 ▶); Michael (2005 ▶); Nadal & Olavarria (2004 ▶).
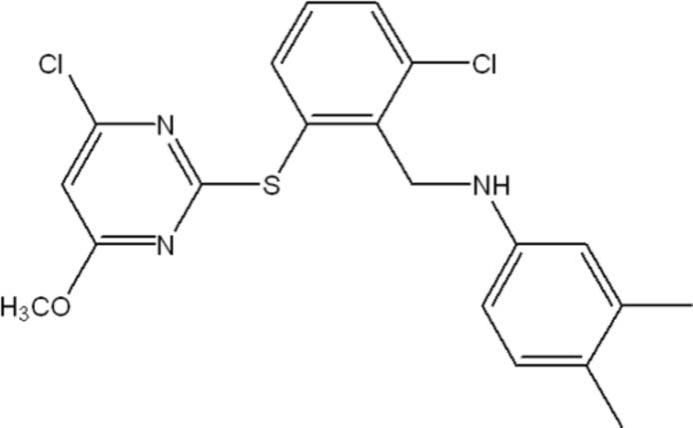


## Experimental

### 

#### Crystal data

C_20_H_19_Cl_2_N_3_OS
*M* *_r_* = 420.34
Monoclinic, 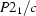

*a* = 12.3653 (12) Å
*b* = 14.1332 (14) Å
*c* = 11.8276 (11) Å
β = 97.340 (1)°
*V* = 2050.1 (3) Å^3^
*Z* = 4
Mo *K*α radiation
μ = 0.43 mm^−1^
*T* = 296 K
0.37 × 0.28 × 0.25 mm

#### Data collection

Bruker APEXII diffractometer
Absorption correction: multi-scan (*SADABS*; Sheldrick, 1996 ▶) *T*_min_ = 0.856, *T*_max_ = 0.899
15364 measured reflections
3804 independent reflections
2727 reflections with *I* > 2σ(*I*)
*R*_int_ = 0.026

#### Refinement

*R*[*F* ^2^ > 2σ(*F* ^2^)] = 0.049
*wR*(*F* ^2^) = 0.139
*S* = 1.04
3804 reflections
235 parameters
H-atom parameters constrained
Δρ_max_ = 0.29 e Å^−3^
Δρ_min_ = −0.48 e Å^−3^

### 

Data collection: *APEX2* (Bruker, 2004 ▶); cell refinement: *SAINT* (Bruker, 2004 ▶); data reduction: *SAINT*; program(s) used to solve structure: *SHELXS97* (Sheldrick, 2008 ▶); program(s) used to refine structure: *SHELXL97* (Sheldrick, 2008 ▶); molecular graphics: *SHELXTL* (Sheldrick, 2008 ▶); software used to prepare material for publication: *SHELXTL*.

## Supplementary Material

Crystal structure: contains datablocks global, I. DOI: 10.1107/S1600536809041026/lh2926sup1.cif

Structure factors: contains datablocks I. DOI: 10.1107/S1600536809041026/lh2926Isup2.hkl

Additional supplementary materials:  crystallographic information; 3D view; checkCIF report

## Figures and Tables

**Table 1:** Hydrogen-bond geometry (Å, °)

*D*—H⋯*A*	*D*—H	H⋯*A*	*D*⋯*A*	*D*—H⋯*A*
N1—H1⋯N3′	0.87	2.31	3.089 (3)	150
N1—H1⋯N2	0.87	2.45	3.203 (4)	145

**Fig. 1.**
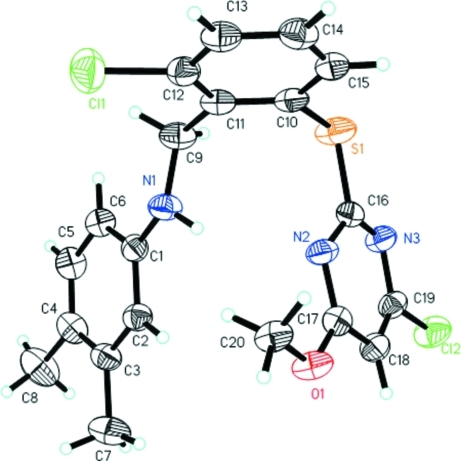
The molecular structure of the title compound with the atom numbering scheme and 30% probability displacement ellipsoids. The disorder is not shown.

**Fig. 2.**
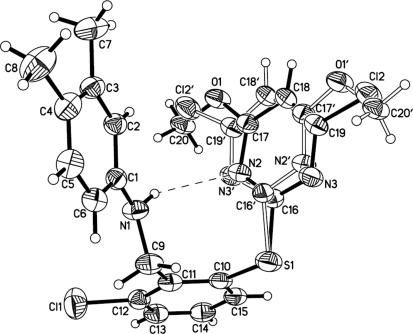
The molecular structure of the title compound with the atom numbering scheme and 30% probability displacement ellipsoids. The minor component of disorder is shown with open bonds and the dashed line represents a hydrogen bond.

## supplementary crystallographic information

### Comment

Pyrimidine derivatives are widespread in medicinal and natural product chemistry. A number of natural products, pharmaceuticals, and functional materials incorporate this heterocycle (Michael, 2005). Several examples of pharmaceutically important compounds include trimethoprim (Joffe *et al.*, 1989), sulfadiazine (Petersen & Schmidt, 2003),Gleevec (imatinib mesilate) (Nadal & Olavarria, 2004), and Xeloda (capecitabine) (Blum, 2001). Natural and unnatural polymers also contain pyrimidine derivatives (Gompper *et al.*, 2004). The potent physiological properties of these pyrimidine derivatives has led to their vast use as medicines in the field of pharmaceutical chemistry. In this context, we report the crystal structure of the title compound.

The molecular structure is shown in Fig. 1. The bond lengths and angles are as expected. The the dihedral angle between the two benzene rings is 79.3 (7)°. The 4-chloro-6-methoxypyrimidine group is rotationlly disordered over two sites by approximately 180° with the ratio of the refined occupancies being 0.6772 (15):0.3228 (15). Both the major and minor components of disorder are involved in intramolecular N-H···N hydrogen bonds.

### Experimental

To a solution of 2,4-dichloro-6-methoxypyrimidine (0.5 mmol) and 2-((3,4-dimethylphenylamino)methyl)-3-chlorobenzenethiol (0.5 mmol) in dry methylbenzene  NaH (0.6 mmol) was added. The mixture was stirred for 12 h at room temperature. After evaporation of the solvent, the residue was purified by column chromatography on silica gel to afford the title compound as a colorless solid (yield 90%). The title compound was recrystallized from CH_2_Cl_2_ at room temperature to give the desired crystals suitable for single-crystal X-ray diffraction.

### Refinement

All H atoms were positioned geometrically and treated as riding, with C—H bond lengths constrained to 0.93 Å (aromatic CH); 0.97 Å (methylene CH_2_);  0.96 Å (methyl), and with *U*ĩso~(H) = 1.2U_eq_(C) or 1.5U_eq_(methyl C).

### Crystal data

**Table:**

C_20_H_19_Cl_2_N_3_OS	*F*(000) = 872
*M**_r_* = 420.34	*D*_x_ = 1.362 Mg m^−^^3^
Monoclinic, *P*2_1_/*c*	Mo *K*α radiation, λ = 0.71073 Å
Hall symbol: -P 2ybc	Cell parameters from 3665 reflections
*a* = 12.3653 (12) Å	θ = 2.7–21.7°
*b* = 14.1332 (14) Å	µ = 0.43 mm^−^^1^
*c* = 11.8276 (11) Å	*T* = 296 K
β = 97.340 (1)°	Block, colourless
*V* = 2050.1 (3) Å^3^	0.37 × 0.28 × 0.25 mm
*Z* = 4	

### Data collection

**Table:**

Bruker APEXII diffractometer	3804 independent reflections
Radiation source: fine-focus sealed tube	2727 reflections with *I* > 2σ(*I*)
graphite	*R*_int_ = 0.026
φ and ω scans	θ_max_ = 25.5°, θ_min_ = 2.3°
Absorption correction: multi-scan (*SADABS*; Sheldrick, 1996)	*h* = −14→14
*T*_min_ = 0.856, *T*_max_ = 0.899	*k* = −17→17
15364 measured reflections	*l* = −14→14

### Refinement

**Table:**

Refinement on *F*^2^	Primary atom site location: structure-invariant direct methods
Least-squares matrix: full	Secondary atom site location: difference Fourier map
*R*[*F*^2^ > 2σ(*F*^2^)] = 0.049	Hydrogen site location: inferred from neighbouring sites
*wR*(*F*^2^) = 0.139	H-atom parameters constrained
*S* = 1.04	*w* = 1/[σ^2^(*F*_o_^2^) + (0.0558*P*)^2^ + 1.1287*P*] where *P* = (*F*_o_^2^ + 2*F*_c_^2^)/3
3804 reflections	(Δ/σ)_max_ = 0.001
235 parameters	Δρ_max_ = 0.29 e Å^−^^3^
0 restraints	Δρ_min_ = −0.48 e Å^−^^3^

### Special details

**Table:**

Geometry. All esds (except the esd in the dihedral angle between two l.s. planes) are estimated using the full covariance matrix. The cell esds are taken into account individually in the estimation of esds in distances, angles and torsion angles; correlations between esds in cell parameters are only used when they are defined by crystal symmetry. An approximate (isotropic) treatment of cell esds is used for estimating esds involving l.s. planes.
Refinement. Refinement of F^2^ against ALL reflections. The weighted R-factor wR and goodness of fit S are based on F^2^, conventional R-factors R are based on F, with F set to zero for negative F^2^. The threshold expression of F^2^ > 2sigma(F^2^) is used only for calculating R-factors(gt) etc. and is not relevant to the choice of reflections for refinement. R-factors based on F^2^ are statistically about twice as large as those based on F, and R- factors based on ALL data will be even larger.

### Fractional atomic coordinates and isotropic or equivalent isotropic displacement parameters (Å^2^)

**Table:**

	*x*	*y*	*z*	*U*_iso_*/*U*_eq_	Occ. (<1)
C16	0.15712 (12)	0.08616 (19)	0.48299 (12)	0.0579 (7)	0.6772 (15)
C17	0.06084 (10)	0.14779 (8)	0.61811 (10)	0.0540 (9)	0.6772 (15)
C18	−0.03179 (7)	0.15182 (8)	0.54001 (8)	0.0582 (11)	0.6772 (15)
H18	−0.0985	0.1704	0.5612	0.070*	0.6772 (15)
C19	−0.02176 (7)	0.12790 (7)	0.43258 (8)	0.0573 (10)	0.6772 (15)
C20	0.15013 (16)	0.16340 (13)	0.81220 (15)	0.0654 (12)	0.6772 (15)
H20A	0.1749	0.0991	0.8113	0.098*	0.6772 (15)
H20B	0.1299	0.1779	0.8860	0.098*	0.6772 (15)
H20C	0.2076	0.2051	0.7962	0.098*	0.6772 (15)
Cl2	−0.13131 (8)	0.13451 (9)	0.32716 (10)	0.0852 (4)	0.6772 (15)
O1	0.05547 (12)	0.17589 (12)	0.72538 (12)	0.0680 (7)	0.6772 (15)
C16'	0.16085 (11)	0.10476 (18)	0.49634 (11)	0.0579 (7)	0.3228 (15)
C19'	0.08829 (11)	0.14785 (9)	0.66157 (11)	0.0573 (10)	0.3228 (15)
C18'	−0.01246 (9)	0.15594 (9)	0.60104 (9)	0.0582 (11)	0.3228 (15)
H18'	−0.0706	0.1800	0.6348	0.070*	0.3228 (15)
C17'	−0.02647 (7)	0.12816 (7)	0.49021 (8)	0.0540 (9)	0.3228 (15)
C20'	−0.11725 (8)	0.11835 (10)	0.30480 (11)	0.0654 (12)	0.3228 (15)
H20D	−0.0699	0.1627	0.2741	0.098*	0.3228 (15)
H20E	−0.1888	0.1221	0.2626	0.098*	0.3228 (15)
H20F	−0.0891	0.0555	0.2991	0.098*	0.3228 (15)
Cl2'	0.11223 (15)	0.18012 (14)	0.80264 (15)	0.0852 (4)	0.3228 (15)
N3'	0.17432 (13)	0.11642 (15)	0.61125 (12)	0.0625 (9)	0.3228 (15)
N2'	0.05936 (9)	0.09825 (12)	0.43865 (9)	0.0551 (8)	0.3228 (15)
O1'	−0.12330 (7)	0.14091 (10)	0.42454 (9)	0.0680 (7)	0.3228 (15)
C1	0.3169 (2)	0.35870 (18)	0.5611 (2)	0.0509 (6)	
C2	0.2300 (2)	0.39912 (19)	0.6075 (2)	0.0554 (6)	
H2	0.1937	0.3634	0.6569	0.067*	
C3	0.1958 (2)	0.4909 (2)	0.5823 (2)	0.0605 (7)	
C4	0.2502 (3)	0.5451 (2)	0.5075 (2)	0.0667 (8)	
C5	0.3344 (3)	0.5040 (2)	0.4610 (2)	0.0682 (8)	
H5	0.3702	0.5392	0.4106	0.082*	
C6	0.3687 (2)	0.4123 (2)	0.4860 (2)	0.0606 (7)	
H6	0.4262	0.3867	0.4525	0.073*	
C7	0.1032 (3)	0.5315 (3)	0.6380 (3)	0.0932 (11)	
H7A	0.0722	0.4828	0.6806	0.140*	
H7B	0.0483	0.5556	0.5805	0.140*	
H7C	0.1299	0.5818	0.6885	0.140*	
C8	0.2164 (4)	0.6463 (2)	0.4800 (3)	0.1028 (13)	
H8A	0.2595	0.6714	0.4250	0.154*	
H8B	0.2276	0.6837	0.5482	0.154*	
H8C	0.1407	0.6479	0.4492	0.154*	
C9	0.4226 (2)	0.2140 (2)	0.5326 (2)	0.0664 (8)	
H9A	0.4922	0.2459	0.5339	0.080*	
H9B	0.3901	0.2079	0.4538	0.080*	
C10	0.3725 (2)	0.0407 (2)	0.5510 (2)	0.0580 (7)	
C11	0.4391 (2)	0.11761 (19)	0.5862 (2)	0.0560 (7)	
C12	0.5198 (2)	0.1017 (2)	0.6773 (2)	0.0591 (7)	
C13	0.5361 (2)	0.0153 (2)	0.7298 (2)	0.0649 (8)	
H13	0.5909	0.0077	0.7906	0.078*	
C14	0.4713 (2)	−0.0591 (2)	0.6921 (3)	0.0668 (8)	
H14	0.4821	−0.1179	0.7268	0.080*	
C15	0.3897 (2)	−0.0469 (2)	0.6025 (3)	0.0655 (8)	
H15	0.3459	−0.0979	0.5764	0.079*	
Cl1	0.60797 (8)	0.19268 (7)	0.72836 (9)	0.1024 (4)	
N1	0.35165 (18)	0.26822 (15)	0.59613 (19)	0.0620 (6)	
H1	0.3029	0.2357	0.6261	0.093*	
N2	0.1576 (3)	0.1175 (2)	0.5905 (3)	0.0551 (8)	0.6772 (15)
N3	0.0724 (3)	0.0956 (3)	0.3984 (3)	0.0625 (9)	0.6772 (15)
S1	0.27000 (6)	0.04895 (7)	0.43144 (7)	0.0834 (3)	

### Atomic displacement parameters (Å^2^)

**Table:**

	*U*^11^	*U*^22^	*U*^33^	*U*^12^	*U*^13^	*U*^23^
C16	0.0475 (15)	0.0469 (17)	0.079 (2)	0.0063 (13)	0.0058 (14)	−0.0074 (15)
C17	0.049 (2)	0.054 (2)	0.059 (2)	0.0051 (17)	0.0072 (18)	0.0076 (17)
C18	0.0427 (19)	0.065 (2)	0.064 (3)	0.0067 (16)	−0.004 (2)	0.000 (2)
C19	0.0444 (19)	0.059 (2)	0.069 (3)	0.0032 (16)	0.0065 (17)	0.0124 (18)
C20	0.049 (2)	0.096 (3)	0.050 (2)	0.019 (2)	0.0033 (17)	0.007 (2)
Cl2	0.0551 (6)	0.1185 (10)	0.0778 (8)	0.0104 (6)	−0.0073 (5)	−0.0032 (7)
O1	0.0512 (14)	0.098 (2)	0.0554 (15)	0.0186 (14)	0.0076 (11)	0.0065 (14)
C16'	0.0475 (15)	0.0469 (17)	0.079 (2)	0.0063 (13)	0.0058 (14)	−0.0074 (15)
C19'	0.0444 (19)	0.059 (2)	0.069 (3)	0.0032 (16)	0.0065 (17)	0.0124 (18)
C18'	0.0427 (19)	0.065 (2)	0.064 (3)	0.0067 (16)	−0.004 (2)	0.000 (2)
C17'	0.049 (2)	0.054 (2)	0.059 (2)	0.0051 (17)	0.0072 (18)	0.0076 (17)
C20'	0.049 (2)	0.096 (3)	0.050 (2)	0.019 (2)	0.0033 (17)	0.007 (2)
Cl2'	0.0551 (6)	0.1185 (10)	0.0778 (8)	0.0104 (6)	−0.0073 (5)	−0.0032 (7)
N3'	0.0452 (18)	0.076 (2)	0.067 (2)	0.0063 (17)	0.0107 (15)	−0.0018 (17)
N2'	0.0462 (17)	0.056 (2)	0.0644 (19)	0.0078 (15)	0.0129 (14)	0.0073 (15)
O1'	0.0512 (14)	0.098 (2)	0.0554 (15)	0.0186 (14)	0.0076 (11)	0.0065 (14)
C1	0.0480 (13)	0.0527 (14)	0.0502 (14)	0.0020 (12)	−0.0013 (11)	−0.0005 (11)
C2	0.0524 (15)	0.0616 (16)	0.0518 (14)	0.0043 (12)	0.0048 (12)	−0.0005 (12)
C3	0.0595 (16)	0.0671 (18)	0.0523 (15)	0.0159 (14)	−0.0028 (13)	−0.0078 (13)
C4	0.084 (2)	0.0598 (17)	0.0520 (15)	0.0133 (15)	−0.0067 (15)	0.0033 (13)
C5	0.081 (2)	0.0665 (19)	0.0563 (16)	−0.0022 (16)	0.0069 (15)	0.0107 (14)
C6	0.0588 (16)	0.0663 (17)	0.0569 (16)	0.0037 (14)	0.0081 (13)	0.0029 (13)
C7	0.096 (3)	0.099 (3)	0.087 (2)	0.039 (2)	0.019 (2)	−0.005 (2)
C8	0.153 (4)	0.068 (2)	0.082 (2)	0.030 (2)	−0.004 (2)	0.0116 (18)
C9	0.0664 (17)	0.0695 (18)	0.0647 (17)	0.0221 (14)	0.0140 (14)	0.0099 (14)
C10	0.0439 (14)	0.0737 (19)	0.0578 (15)	0.0164 (13)	0.0118 (12)	−0.0061 (14)
C11	0.0530 (15)	0.0644 (17)	0.0522 (15)	0.0182 (13)	0.0124 (12)	0.0033 (12)
C12	0.0542 (15)	0.0612 (17)	0.0615 (16)	0.0092 (13)	0.0058 (12)	−0.0004 (13)
C13	0.0612 (17)	0.077 (2)	0.0569 (16)	0.0209 (15)	0.0070 (13)	0.0076 (15)
C14	0.0713 (19)	0.0633 (18)	0.0693 (18)	0.0155 (15)	0.0227 (15)	0.0130 (15)
C15	0.0545 (16)	0.0673 (19)	0.079 (2)	0.0039 (14)	0.0258 (15)	−0.0090 (15)
Cl1	0.0947 (7)	0.0804 (6)	0.1220 (8)	−0.0042 (5)	−0.0248 (6)	−0.0057 (5)
N1	0.0617 (14)	0.0545 (13)	0.0732 (15)	0.0142 (11)	0.0217 (12)	0.0084 (11)
N2	0.0462 (17)	0.056 (2)	0.0644 (19)	0.0078 (15)	0.0129 (14)	0.0073 (15)
N3	0.0452 (18)	0.076 (2)	0.067 (2)	0.0063 (17)	0.0107 (15)	−0.0018 (17)
S1	0.0551 (4)	0.1282 (8)	0.0650 (5)	0.0243 (5)	0.0012 (4)	−0.0217 (5)

### Geometric parameters (Å, °)

**Table:**

C16—N2	1.345 (4)	C2—C3	1.385 (4)
C16—N3	1.360 (4)	C2—H2	0.9300
C16—S1	1.6775 (16)	C3—C4	1.405 (4)
C17—O1	1.3392	C3—C7	1.505 (4)
C17—N2	1.350 (3)	C4—C5	1.368 (4)
C17—C18	1.3777	C4—C8	1.513 (4)
C18—C19	1.3358	C5—C6	1.385 (4)
C18—H18	0.9300	C5—H5	0.9300
C19—N3	1.360 (3)	C6—H6	0.9300
C19—Cl2	1.7224	C7—H7A	0.9600
C20—O1	1.4659	C7—H7B	0.9600
C20—H20A	0.9600	C7—H7C	0.9600
C20—H20B	0.9600	C8—H8A	0.9600
C20—H20C	0.9600	C8—H8B	0.9600
C16'—N2'	1.3529	C8—H8C	0.9600
C16'—N3'	1.3581	C9—N1	1.446 (3)
C16'—S1	1.8159 (16)	C9—C11	1.505 (4)
C19'—N3'	1.3582	C9—H9A	0.9700
C19'—C18'	1.3601	C9—H9B	0.9700
C19'—Cl2'	1.7185	C10—C15	1.384 (4)
C18'—C17'	1.3581	C10—C11	1.395 (4)
C18'—H18'	0.9300	C10—S1	1.778 (3)
C17'—O1'	1.3535	C11—C12	1.390 (4)
C17'—N2'	1.3573	C12—C13	1.373 (4)
C20'—O1'	1.4629	C12—Cl1	1.743 (3)
C20'—H20D	0.9600	C13—C14	1.362 (4)
C20'—H20E	0.9600	C13—H13	0.9300
C20'—H20F	0.9600	C14—C15	1.377 (4)
C1—C6	1.384 (4)	C14—H14	0.9300
C1—C2	1.390 (3)	C15—H15	0.9300
C1—N1	1.395 (3)	N1—H1	0.8684
			
N2—C16—N3	125.0 (2)	C6—C5—H5	118.6
N2—C16—S1	122.89 (17)	C1—C6—C5	119.7 (3)
N3—C16—S1	111.19 (16)	C1—C6—H6	120.2
O1—C17—N2	118.44 (16)	C5—C6—H6	120.2
O1—C17—C18	119.0	C3—C7—H7A	109.5
N2—C17—C18	122.52 (16)	C3—C7—H7B	109.5
C19—C18—C17	117.2	H7A—C7—H7B	109.5
C19—C18—H18	121.4	C3—C7—H7C	109.5
C17—C18—H18	121.4	H7A—C7—H7C	109.5
C18—C19—N3	123.72 (16)	H7B—C7—H7C	109.5
C18—C19—Cl2	121.0	C4—C8—H8A	109.5
N3—C19—Cl2	115.28 (16)	C4—C8—H8B	109.5
C17—O1—C20	119.8	H8A—C8—H8B	109.5
N2'—C16'—N3'	120.0	C4—C8—H8C	109.5
N2'—C16'—S1	116.68 (7)	H8A—C8—H8C	109.5
N3'—C16'—S1	118.23 (6)	H8B—C8—H8C	109.5
N3'—C19'—C18'	120.9	N1—C9—C11	108.6 (2)
N3'—C19'—Cl2'	117.7	N1—C9—H9A	110.0
C18'—C19'—Cl2'	121.4	C11—C9—H9A	110.0
C17'—C18'—C19'	118.6	N1—C9—H9B	110.0
C17'—C18'—H18'	120.7	C11—C9—H9B	110.0
C19'—C18'—H18'	120.7	H9A—C9—H9B	108.3
O1'—C17'—N2'	118.0	C15—C10—C11	121.0 (3)
O1'—C17'—C18'	120.6	C15—C10—S1	117.5 (2)
N2'—C17'—C18'	120.9	C11—C10—S1	121.3 (2)
O1'—C20'—H20D	109.5	C12—C11—C10	116.3 (2)
O1'—C20'—H20E	109.5	C12—C11—C9	121.2 (3)
H20D—C20'—H20E	109.5	C10—C11—C9	122.5 (2)
O1'—C20'—H20F	109.5	C13—C12—C11	122.9 (3)
H20D—C20'—H20F	109.5	C13—C12—Cl1	116.8 (2)
H20E—C20'—H20F	109.5	C11—C12—Cl1	120.3 (2)
C16'—N3'—C19'	118.4	C14—C13—C12	119.5 (3)
C16'—N2'—C17'	118.7	C14—C13—H13	120.2
C17'—O1'—C20'	112.1	C12—C13—H13	120.2
C6—C1—C2	118.2 (2)	C13—C14—C15	119.9 (3)
C6—C1—N1	122.8 (2)	C13—C14—H14	120.1
C2—C1—N1	118.9 (2)	C15—C14—H14	120.1
C3—C2—C1	122.0 (3)	C14—C15—C10	120.4 (3)
C3—C2—H2	119.0	C14—C15—H15	119.8
C1—C2—H2	119.0	C10—C15—H15	119.8
C2—C3—C4	119.2 (3)	C1—N1—C9	121.0 (2)
C2—C3—C7	119.4 (3)	C1—N1—H1	113.8
C4—C3—C7	121.3 (3)	C9—N1—H1	115.5
C5—C4—C3	118.1 (3)	C16—N2—C17	115.8 (3)
C5—C4—C8	121.2 (3)	C16—N3—C19	115.0 (3)
C3—C4—C8	120.6 (3)	C16—S1—C10	105.87 (10)
C4—C5—C6	122.7 (3)	C16—S1—C16'	9.0
C4—C5—H5	118.6	C10—S1—C16'	100.69 (9)
			
O1—C17—C18—C19	176.6	S1—C10—C11—C9	5.4 (3)
N2—C17—C18—C19	−2.7 (2)	N1—C9—C11—C12	−85.8 (3)
C17—C18—C19—N3	3.5 (2)	N1—C9—C11—C10	91.6 (3)
C17—C18—C19—Cl2	−177.7	C10—C11—C12—C13	1.2 (4)
N2—C17—O1—C20	−6.7 (2)	C9—C11—C12—C13	178.7 (3)
C18—C17—O1—C20	174.0	C10—C11—C12—Cl1	179.91 (19)
N3'—C19'—C18'—C17'	2.7	C9—C11—C12—Cl1	−2.5 (3)
Cl2'—C19'—C18'—C17'	−179.2	C11—C12—C13—C14	0.2 (4)
C19'—C18'—C17'—O1'	−175.4	Cl1—C12—C13—C14	−178.6 (2)
C19'—C18'—C17'—N2'	−3.9	C12—C13—C14—C15	−0.5 (4)
N2'—C16'—N3'—C19'	−18.4	C13—C14—C15—C10	−0.5 (4)
S1—C16'—N3'—C19'	−172.43 (17)	C11—C10—C15—C14	1.9 (4)
C18'—C19'—N3'—C16'	8.3	S1—C10—C15—C14	177.0 (2)
Cl2'—C19'—N3'—C16'	−169.9	C6—C1—N1—C9	−18.3 (4)
N3'—C16'—N2'—C17'	17.4	C2—C1—N1—C9	165.0 (3)
S1—C16'—N2'—C17'	171.73 (17)	C11—C9—N1—C1	−176.0 (2)
O1'—C17'—N2'—C16'	165.7	N3—C16—N2—C17	10.0 (6)
C18'—C17'—N2'—C16'	−6.0	S1—C16—N2—C17	177.98 (17)
N2'—C17'—O1'—C20'	0.4	O1—C17—N2—C16	177.1 (2)
C18'—C17'—O1'—C20'	172.2	C18—C17—N2—C16	−3.6 (4)
C6—C1—C2—C3	−1.2 (4)	N2—C16—N3—C19	−9.2 (6)
N1—C1—C2—C3	175.7 (2)	S1—C16—N3—C19	−178.4 (2)
C1—C2—C3—C4	0.1 (4)	C18—C19—N3—C16	2.0 (4)
C1—C2—C3—C7	−178.3 (3)	Cl2—C19—N3—C16	−176.9 (2)
C2—C3—C4—C5	0.9 (4)	N2—C16—S1—C10	10.2 (3)
C7—C3—C4—C5	179.2 (3)	N3—C16—S1—C10	179.7 (3)
C2—C3—C4—C8	−178.8 (3)	N2—C16—S1—C16'	−45.6 (3)
C7—C3—C4—C8	−0.4 (4)	N3—C16—S1—C16'	123.9 (3)
C3—C4—C5—C6	−0.9 (4)	C15—C10—S1—C16	95.5 (2)
C8—C4—C5—C6	178.8 (3)	C11—C10—S1—C16	−89.4 (2)
C2—C1—C6—C5	1.2 (4)	C15—C10—S1—C16'	103.1 (2)
N1—C1—C6—C5	−175.5 (3)	C11—C10—S1—C16'	−81.9 (2)
C4—C5—C6—C1	−0.2 (4)	N2'—C16'—S1—C16	−35.93 (14)
C15—C10—C11—C12	−2.2 (4)	N3'—C16'—S1—C16	118.9
S1—C10—C11—C12	−177.06 (19)	N2'—C16'—S1—C10	−161.89 (12)
C15—C10—C11—C9	−179.7 (2)	N3'—C16'—S1—C10	−7.04 (15)

### Hydrogen-bond geometry (Å, °)

**Table:**

*D*—H···*A*	*D*—H	H···*A*	*D*···*A*	*D*—H···*A*
N1—H1···N3'	0.87	2.31	3.089 (3)	150
N1—H1···N2	0.87	2.45	3.203 (4)	145
